# Exercise Behavior and Mood during the COVID-19 Pandemic in Taiwan: Lessons for the Future

**DOI:** 10.3390/ijerph17197092

**Published:** 2020-09-28

**Authors:** Yu-Kai Chang, Chiao-Ling Hung, Sinika Timme, Sanaz Nosrat, Chien-Heng Chu

**Affiliations:** 1 Department of Physical Education, National Taiwan Normal University, Taipei 10617, Taiwan; yukaichangnew@gmail.com; 2 Institute for Research Excellence in Learning Science, National Taiwan Normal University, Taipei 10617, Taiwan; 3 Department of Athletics, National Taiwan University, Taipei 10617, Taiwan; musehung@gmail.com; 4 Sport and Exercise Psychology, University of Potsdam, 14469 Potsdam, Germany; 5 Department of Health Sciences, Lehman College, Bronx, NY 10468, USA; sanaz.nosrat@lehman.cuny.edu

**Keywords:** cumulative link model, exercise frequency, physical activity, POMS, social restriction, stress

## Abstract

The Coronavirus disease 2019 (COVID-19) pandemic and its associated governmental recommendations and restrictions have influenced many aspects of human life, including exercise and mental health. This study aims to explore the influence of COVID-19 on exercise behavior and its impact on mood states, as well as predict changes in exercise behavior during a similar future pandemic in Taiwan. A cross-sectional online survey was conducted between 7 April and 13 May 2020 (*n* = 1114). Data on exercise behavior pre and during the pandemic and mood states were collected. A cumulative link model was used to predict changes in exercise frequency during a similar future pandemic by exercise frequency during the pandemic. A linear model was used to predict the influence of exercise frequency before and during the pandemic on mood states during the pandemic. A total of 71.2%, 67.3%, and 58.3% of respondents maintained their exercise intensity, frequency, and duration, respectively, during the pandemic. Frequent exercisers are more likely to maintain their exercise frequency during a similar pandemic (*p* < 0.001). Higher exercise frequencies during the pandemic were associated with better mood states (*p* < 0.05). Moreover, the effects of prepandemic exercise frequency on mood states are moderated by changes in exercise frequency during the pandemic (*p* < 0.05). Additionally, maintenance of exercise frequency during a pandemic specifically for frequent exercisers are recommended to preserve mood states. These results may provide evidence for health policies on exercise promotion and mental health before and during a future pandemic.

## 1. Introduction

Coronavirus disease 2019 (COVID-19), generated by severe acute respiratory syndrome coronavirus 2 (SARS-CoV-2) [1], has spread rapidly and globally, with 12,552,765 confirmed cases and 561,617 deaths reported worldwide as of 12 July 2020 [2] after the first case reported in Wuhan, China in December 2019 [3]. This crisis, yet, has affected each country differently. Taiwan was anticipated to have the second-highest number of cases because of proximity and frequent travels to and from China [4]. However, learning from severe acute respiratory syndrome (SARS), Taiwan took speedy responses, proactive deployments, as well as novel strategies to identify and contain the COVID-19 [4,5,6,7], resulting in only 443 cases and seven deaths as of 7 June 2020 which is the official date Taiwan loosened COVID-19 measures. It is worth mentioning that Taiwan did not enforce any lockdown measures similar to other governments around the world.

The COVID-19 pandemic has led to numerous behavior changes such as social distancing, staying at home, avoiding crowds, or avoiding the usual venues of exercise such as gyms and fitness clubs [2,8]. Therefore, this pandemic has created a special situation for researchers to examine changes in exercise behavior. In Taiwan specifically, in order to suppress the transmission, recommendations included strict social distancing, self-isolation, quarantine, mandatory wearing of medical face masks in public, measuring body temperature at all entrances, and restricting the number of people in the entertainment venues [9], which may have created a novel and unique barrier to maintaining exercise behavior among Taiwanese.

In addition to this novel situation created by the pandemic, the physiological and psychological benefits of exercise are other important reasons to investigate exercise behavior changes at this time. Regular exercise of moderate intensity is positively linked to immune function [10] and reduction in respiratory symptoms [11], the dominant clinical manifestation of COVID-19 [12,13]. The beneficial effects on exercise may also lead to a lower COVID-19 mortality rate due to its effect on COVID-19 related cardiovascular complications (e.g., cardiovascular disease and myocardial injury) and its comorbidities (e.g., hypertension, type-2 diabetes, and obesity) [14], which are recognized as critical risk factors of COVID-19 [13,15]. Additionally, physical inactivity significantly elevates the risk of COVID-19 even after adjusting for age, sex, and other lifestyle factors (e.g., smoking and alcohol intake) [16]. Exercise is also linked to better mood states and better subjective well-being [17]. A large-scale nationwide study conducted in the United States has shown a negative association between exercise levels and self-reported mental health burdens such as stress, depression, and problems with emotions [18]. Additionally, exercise termination has been shown to have adverse consequences for mental health [19]. These studies provide indirect evidence for the potential role of exercise on mental health during this pandemic. This is specifically important as the COVID-19 pandemic is linked to many psychological adverse effects [20,21,22,23]. For example, the general population might become anxious and fearful due to the high transmission rate of COVID-19 [24]; healthcare providers might experience stress, anxiety, or insomnia due to the high susceptibility to COVID-19 [25]; those who have a positive diagnosis, might encounter discrimination or emotional isolation [26], and the survivors might develop anxiety, depression, and post-traumatic stress disorder [27].

It is evident that exploring exercise behavior changes and its potential consequences on mental health during the COVID-19 pandemic is essential. Additionally, how the COVID-19 pandemic and future similar pandemics affect exercise behavior and mental health in Taiwan remain unknown. This study aims to determine whether and how COVID-19 has affected exercise behavior and mental health status in Taiwan. Additionally, a model was established to predict the changes in exercise behavior during similar future pandemics, and potential moderators (e.g., age, education, and gender) were examined.

## 2. Methods

### 2.1. Study Design

The study used a cross-sectional design to investigate changes in exercise behavior and its relation to changes in mood states during the COVID-19 pandemic in Taiwan. This study is part of a larger project that was initiated in Potsdam Germany by the “International Research Group on COVID and exercise (International Research Group (IRG) on COVID and exercise)” and was translated into 18 languages. Overall, 16,353 respondents from 99 countries participated in this study (i.e., completed the questionnaire) [28]. This study is specifically the report of the results from Taiwan. Data were collected with an online survey using the Unipark™ web-based survey software. The study was conducted from April 7 to 13 May 2020, and the questionnaires were translated into traditional Chinese for use in Taiwan. General Data Protection Regulations (EU) and compliance with the APA Ethical Guidelines for Research were fulfilled and were also acknowledged by the participants. Ethical approval was waived for this study as the survey was completely anonymous.

### 2.2. Participants

Participants were recruited through social media, including Facebook, Line, Instagram, Twitter, as well as personal referrals. We did not use any screening questionnaire for underlying psychological conditions to exclude respondents from participating in the survey. No statistical method was used to predetermine the sample size and as many participants as possible were sampled during the time period of global lockdown. A post-hoc power analysis revealed that our sample (*n* = 1104) corresponds to the recommendation for sufficient power in linear models [29]. All participants completed an informed consent before participating in the survey. The participants did not receive any incentive for their participation. Additionally, they were able to skip any questions they did not want to answer or stop answering all questions at any point in time. We collected information on the presence of COVID-19 symptoms or a positive diagnosis to exclude these individuals from the analysis.

### 2.3. Variables

#### 2.3.1. Demographics

Information on age, gender, educational level, income level, and living environment was collected. Income was measured with the question “compared with the average income in your country, which one is your household income?” For statistical analysis, the answers “I don’t have an income at the moment”, “very low income”, and “low income” were combined as “low income”, “high income”, and “very high income” were combined as “high income”, and “medium income” stayed the same.

In order to demonstrate the unique influence of COVID-19 on human behaviors in Taiwan, the presence of governmental pandemic control strategies (i.e., restrictions and recommendations) and the status of recreational facilities (e.g., gyms, clubs, and outdoor facilities) internationally were compared. Additionally, participants’ compliance with those regulations were presented.

#### 2.3.2. Exercise Behavior

Exercise frequency during the COVID-19 pandemic was measured with one question on how often people exercised during the pandemic. We defined exercise for participants as any activity they chose to do as their exercise (e.g., workouts at home, running outside, etc...). Participants were also informed that any physical activity as part of their occupation must not be included unless they are a professional fitness coach or have a similar profession. For statistical analysis, the answers “never”, “once in a while” and “1 day per week” were combined as “1 day or less”, the answers “2 days per week” and “3 days per week” were combined as “2–3 days per week”, and “4–5 days per week”, and “6 days per week”, and “every day” answers were combined as “4 days per week or more”. Exercise frequency before the COVID-19 pandemic was measured and processed in the same format. Participants were also asked about their exercise intensity both during and prepandemic, and they could respond by choosing “low”, “moderate”, “high”, or “very high” intensity. The options “high” and “very high” intensities were combined as “high intensity” for the analysis. Participants were also asked about their exercise session length during the COVID-19 pandemic compared to prepandemic and could choose between “shorter”, “longer”, or “they were of about the same duration”.

#### 2.3.3. Mood

Mood was measured with 16 items from the Profile of Mood States (POMS; [30]). The POMS is a heavily used psychometric questionnaire that measures general well-being in the clinical field both with the general population and people with chronic disease, as well as in sport and exercise psychology research [31]. In this study, participants were asked to report how they felt in the last few days during the pandemic.

For this study, we used the 16-item POMS from a German short screening version, which was psychometrically tested using data from a large national and representative sample [32]. The German items were then matched with the English originals as thoroughly as possible, and then translated from English into traditional Chinese by the research group in Taiwan. These 16 items allow subscores for “depression/anxiety”, “vigor”, “fatigue”, and “irritability”; however, we only used the total score in our analysis. The higher values on POMS indicate more positive mood states. In our study, the 16-item POMS total score achieved an internal consistency (reliability) of Cronbach’s *α* = 0.88. Mean total scores were calculated if at least 10 items of the scale were answered by the participants.

### 2.4. Statistical Analysis

Cumulative link models (CLM) were employed to analyze which variables were significant predictors of the exercise frequency during a similar pandemic condition. Three levels of exercise frequency per week during the pandemic (i.e., “1 day or less”, “2–3 days”, “4 or more days”) were predicted by three levels of exercise frequency per week prepandemic (i.e., “1 day or less”, “2–3 days”, “4 or more days”) with covariates such as age, gender, and income.

A linear model with a priori contrasts was used to analyze the influence of exercise behavior on mood states. This model was run with mood as the numerical response variable and “prepandemic exercise frequency” and “exercise frequency during the pandemic” as categorical predictors (with three levels of “1 day or less”, “2–3 days”, “4 or more days”). We specified a priori contrasts to compare mood scores of different levels of exercise frequency pre and during the COVID-19 pandemic, with exercising “1 day or less” as the intercept. The significance level was set to α = 0.05.

All analyses were performed with R software [33] and the ordinal package [34] for cumulative link models.

## 3. Results

### 3.1. Demographics

A total of 1174 participants filled out the questionnaire in Taiwan. We excluded participants who reported either presence of COVID-19 symptoms or diagnosis at the time of this study (*n* = 60). Therefore, a total of 1114 participants (*mean* (*M*)_age_ = 35.90, standard deviation (*SD*)_age_ = 15.16, 53.9% female) were included in the analysis. Descriptive statistics of the participants, including gender distributions, age, educational level, living environment, and income, are summarized in Table 1.

Participants were also asked about the presence of social restrictions and recommendations and their level of compliance. Although participants reported almost no formal restrictions (92.4%), 81.8% reported that there were recommendations on social distancing, and the majority (86.1%) stated that they did their best to follow these recommendations. This is in contrast to the international data of the IRG on COVID and exercise project, indicating that the majority of participants (79.7%) reported the presence of strict formal restrictions by their governments.

Participants were also asked about the status of the recreational facilities (e.g., gyms, clubs, outdoor facilities, and parks) where they live. A total of 34.2% reported that the gyms and clubs were closed and only 9.5% reported that the outdoor facilities and parks were closed. This is in contrast with the international data indicating 91.1% reported closures of gyms and clubs, and 76.3% reported closures of outdoor facilities and parks. Table 2 provides more detail on this information in Taiwan and a comparison with the international data.

### 3.2. Exercise Behavior

#### 3.2.1. Exercise Behavior Change during the COVID-19 Pandemic

The results show that 67.3% of participants reported the same exercise frequency, 19.7% reported a decrease in exercise frequency, and 12.9% reported an increase in exercise frequency during the COVID-19 pandemic compared to prepandemic.

**Figure 1:**
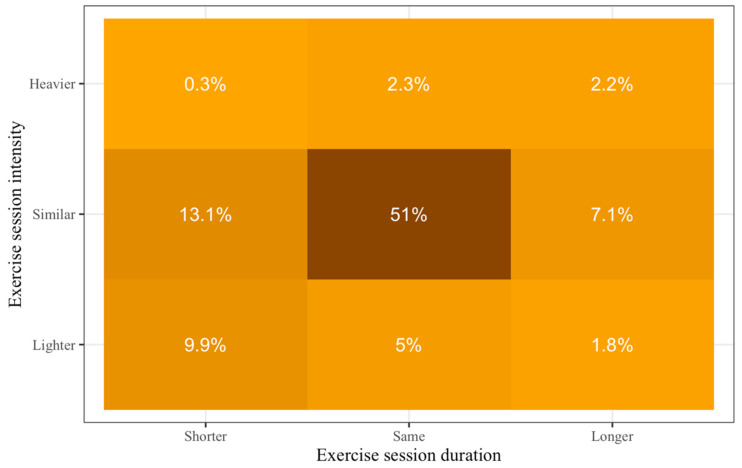
Changes in exercise levels during the Coronavirus disease 2019 (COVID-19) pandemic compared to prepandemic. Darker colors show higher percentages.

Of those who exercised during the COVID-19 pandemic, 71.2% reported being physically active at similar, 16.7% at lighter, and 4.8% at higher exercise intensities. Additionally, 58.3% reported the same exercise duration, 23.3% reported shorter, and 11.1% reported longer exercise duration. This information is presented in Figure 1 in more detail.

#### 3.2.2. Exercise Behavior Change Predictions

A CLM was employed to predict changes in exercise frequency during a pandemic from prepandemic exercise frequency for use in future conditions similar to the current pandemic. The results show that those who exercise 2–3 days per week before a similar pandemic have a significantly higher probability of maintaining their exercise frequency or do more during such pandemics compared to people who exercise one day or less before a similar pandemic (beta coefficient (*b*)_pre2-1_ = 1.95, *p* < 0.001). Those who exercise four days or more per week before a similar pandemic have a significantly higher probability of maintaining their exercise frequency or do more during such pandemics compared to those who exercise 2–3 days per week before a similar pandemic (*b*_pre3-2_ = 3.01, *p* < 0.001). Prepandemic exercise behavior could explain 56.4% of the variance in exercise behavior during a pandemic (R^2^_Nagelkerke_).

**Figure 2:**
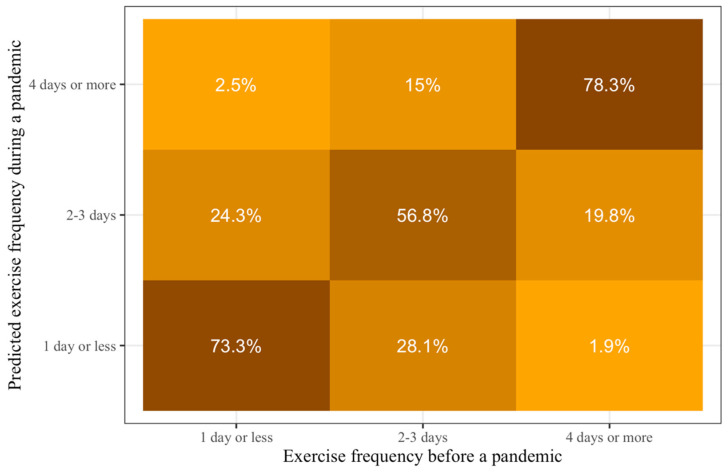
Predictions of probabilities of exercise frequency during conditions similar to COVID-19 pandemic based on prepandemic exercise frequency. Lighter colors show smaller probabilities and darker colors show larger probabilities. The darkest colors are all on the diagonal from the bottom left to the top right, which means that people who exercise at a specific frequency before such pandemics would be most likely to exercise at the same frequency during it.

Calculating the category probabilities from the models’ prediction and location coefficients, we can see that the majority of the Taiwanese will maintain their prepandemic exercise frequency during similar pandemics (Figure 2). Specifically, the probabilities of maintaining exercise frequency during a similar pandemic for those who exercise one day or less per week, 2–3 days per week, and four days or more per week, are 73.3%, 56.8%, and 78.3%, respectively.

We also included gender, age, education, and income as covariates in separate models to predict exercise behavior during similar future pandemics. The results show that there was a main effect of age (*b*_age_ = 0.01, *p* < 0.01) for exercise frequency during a pandemic. This means that older individuals are more likely to have higher exercise frequency during similar pandemics compared to younger individuals.

There were no significant main effects of gender or education. However, levels of these covariates showed significant interaction effects, meaning that the relationship between exercise behavior before a pandemic and exercise behavior during a pandemic is different for specific predictor levels. Specifically, females who exercise one day or less before a pandemic are more likely to stay inactive compared to others (*b*_pre2-1*Female_ = 0.63, *p* = 0.03). Additionally, those who have “completed vocational school or college” and exercise one day or less before a pandemic are more likely to increase their exercise frequency during similar pandemics (*b*_pre2-1*Education4_ = –0.88, *p* < 0.01) compared to other respondents.

Finally, income was a significant predictor of exercise frequency during similar pandemics with those reporting a high level of income (compared to the average income level) being more likely to have higher exercise frequency compared to those with low levels of income (*b*_IncomeHigh_ = 0.43, *p* = 0.03). However, when taking prepandemic exercise frequency into account, income is no longer a significant predictor of exercise frequency during a pandemic (*b*_IncomeHigh_ = 0.23, *p* = 0.37).

Importantly, prepandemic exercise frequency remained a significant predictor of exercise frequency in all models when controlling for possible covariates. Complete statistical results can be seen in Table 3.

### 3.3. Exercise and Mood

**Figure 3:**
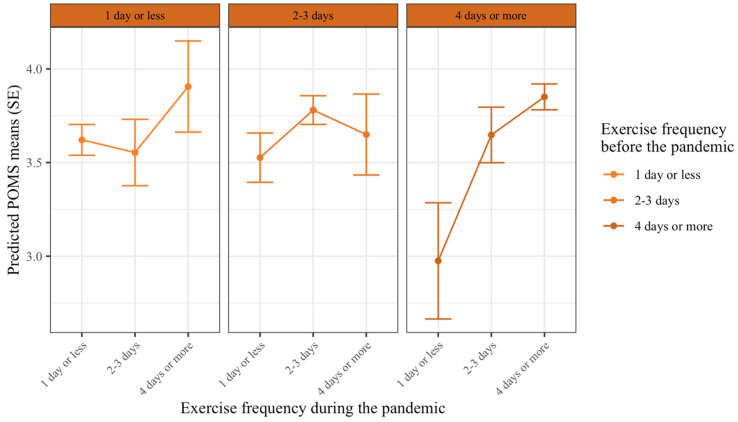
The effect of exercise frequency during the COVID-19 pandemic on mood states depending on prepandemic exercise frequency. Lines indicate values for mood during the pandemic, (higher values are better mood states). Each column indicates exercise frequency before the pandemic, and exercise frequency levels within each column are exercise frequency levels during the pandemic. Error bars indicate 95% confidence intervals. There was a significant difference in mood for those who exercised 4 days or more before the pandemic (right columns) and decreased their exercise frequency during the pandemic. For those, who exercised for 2–3 days before the pandemic (middle column), only those who exercised 1 day or less reported significantly lower mood than those who maintained their exercise frequency.

In this analysis, the mood state was predicted by both prepandemic exercise frequency and exercise frequency during the COVID-19 pandemic. The results show that there was a significant main effect of exercise frequency during the pandemic on mood states. Those who exercised four days or more had significantly higher mood states compared to those who exercised for 2–3 days (*b*_during3-2_ = 0.14, *p* = 0.04), and those exercised for 2–3 days had significantly higher mood states compared to those who exercised one day or less per week during the pandemic (*b*_during2-1_ = 0.29, *p* < 0.001). There was also a significant main effect of prepandemic exercise frequency on mood states. Specifically, those who exercised four days or more per week prepandemic had a significantly lower mood state during the pandemic, compared to those who exercised for 2–3 days per week prepandemic (*b*_pre3-2_ = −0.16, *p* = 0.03). However, there was a significant interaction effect on exercise frequency levels during the pandemic × prepandemic exercise frequency levels on mood (*b*_pre*during_ = −0.48–0.42, *p* = 0.01–0.03). Meaning, the effects of prepandemic exercise frequency on mood were dependent on exercise frequency during the pandemic (Figure 3). Table 4 summarizes the complete statistical results.

Along with post-hoc contrasts (see Table 5), Figure 3 shows that those who exercised four days or more before the COVID-19 pandemic and decreased their exercise frequency during the pandemic experienced a decline in their mood states. Specifically, individuals who decreased their exercise frequency to 2–3 days per week, had significantly lower mood states than those who maintained their exercise frequency (*b*_pre3: during2-3_ = −0.20, *p* = 0.01), and if they decreased their exercise frequency to one day or less per week, they experienced even a more significant decline in mood states (*b*_pre3: during1-3_ = −0.88, *p* < 0.001).

Those who exercised for 2–3 days per week prepandemic and were able to maintain their exercise frequency, had higher mood states compared to those who decreased their exercise frequency to one day or less during the COVID-19 pandemic (*b*_pre2:during1-2_ = −0.25, *p* < 0.01). Exercise frequency during the COVID-19 pandemic had no significant effect on mood states for those who exercised one day or less prepandemic (*p* > 0.05). Overall, exercise behavior could explain 5.2% of the variability in mood states.

## 4. Discussion

The study presents the data from a larger study, “IRG on COVID and exercise”, to examine changes in exercise behavior and its relation to mood in Taiwan during the COVID-19 pandemic.

Our results showed that the majority of respondents were able to maintain their exercise behavior during this pandemic. Prediction analysis further revealed that Taiwanese are likely to maintain their prepandemic exercise frequency during future similar pandemics. Additionally, those who exercise more frequently before a similar pandemic have higher probabilities of maintaining their exercise frequency during such pandemics. Notably, individuals who “completed vocational school or college”, and are rarely active (i.e., exercise one day or less) before a pandemic are more likely to increase their exercise frequency during a future pandemic, while females who are rarely active (i.e., exercise one day or less) before a pandemic tend to maintain their exercise frequency during a future pandemic. Additionally, it seems that older individuals and those with higher levels of income are more likely to engage in higher exercise frequencies.

The relationship between exercise frequency and mood during the COVID-19 pandemic was dependent on the change in exercise behavior (before vs. during). In general, higher frequencies of exercise during the COVID-19 pandemic resulted in better mood states. Additionally, exercising “2–3 days” or “four days or more” before a pandemic was associated with worse mood states if these individuals reduced their exercise frequency during the pandemic.

### 4.1. Exercise Behavior before and during a Pandemic

Generally, a decline in exercise levels is expected during the COVID-19 pandemic and our study indicated that nearly 20% of individuals decreased their exercise frequency. However, our results are inconsistent with previous studies showing a dramatic decrease in physical activity during the COVID-19 pandemic in other parts of the world [8,23,35]; that is, the majority of Taiwanese maintained their exercise frequency, duration, or intensity during the COVID-19 pandemic. These differences may be partially the result of a relatively safe living environment and Taiwanese self-discipline in epidemic prevention. Given the geographical proximity to and the number of visits from mainland China, Taiwan Central Epidemic Control Center (CECC) of Taiwan Centers for Disease Control has been on constant alert about the epidemic in China. After the initial suspicious unknown acute respiratory syndrome case reported in December 2019 in China [3], Taiwan CECC has quickly mobilized and established comprehensive and proactive deployments to counteract and reduce the transmission of COVID-19. These included air and sea border control (e.g., assessing passengers for fever and pneumonia symptoms and restrictions at entries of international and cross-strait ports), case identification and containment (e.g., rapid screening tests for COVID-19, digital contact tracing, and quarantining suspicious cases), increase production rates and control of the domestic market price of medical face masks, and measuring body temperature at all entrances [4,5,6,7,36]. Furthermore, a high percentage of Taiwanese (86.1%) reported that they were willing to comply with Taiwan CECC derived public propaganda and school education for COVID-19 (e.g., maintaining social distance, regular disinfected living areas, and wearing medical face masks in public places) during the early days of the outbreak, which further decreased the spread of COVID-19. Therefore, Taiwan remained free from lockdown restrictions and kept the majority of health and fitness gyms and outdoor recreation facilities (e.g., parks, and playgrounds) open, and was able to maintain social activities per usual which reflect the successful containment of COVID-19.

The prediction analysis demonstrates that exercise frequency during the pandemic is dependent on prepandemic exercise frequency. Specifically, those who are more frequent exercisers prepandemic are more likely to stay active during a similar future pandemic. This finding reflects the importance of “prevention is better than cure” for exercise behavior during a future pandemic (e.g., second wave of the COVID-19 pandemic). Notably, some demographic variables might moderate exercise behavior. Specifically, older individuals are more likely to have higher exercise frequency during similar pandemics. The result is similar to other studies showing that older adults in Taiwan and mainland China exercise more regularly compared to younger individuals [35,37]. Additionally, our data show that inactive individuals who have “completed vocational school or college” are more likely to increase their activity levels compared to the rest of our sample. This is not surprising as generally, individuals with higher educational levels might have better financial resources [38] and more access to health information [39,40]. Finally, we observed that females who are rarely active are more likely to maintain their low levels of exercise frequency. A lower prevalence of physical activity among females compared to males has been previously reported [41,42]. It is plausible that females, in general, perceive more barriers to exercise, such as lack of time due to multitasking (e.g., requiring to take care of others while working) and therefore, they are less likely to change their exercise behavior during such pandemics [43,44]. Collectively, given that age, education, and gender can influence exercise behavior, specific strategies are required to consider and implement for these populations.

Interestingly, approximately 13% of participants reported an increase in exercise frequency and the prediction analysis showed that 26.8% of the individuals who exercise one day or less per week, and 15% of individuals who exercise 2–3 days per week, might increase their exercise frequencies during a future similar pandemic. This shows that inactive or rarely active individuals might increase their exercise frequency or adopt exercise behavior during a pandemic. Our finding is similar to data that showed increased health-seeking behavior (e.g., spending more time exercising) after the SARS epidemic in Hong Kong [45]. Additionally, the perceived severity and susceptibility to disease is associated with both increased likelihoods of conducting health-seeking behavior according to the Health Belief Model [46], and moving to the higher stages of the Transtheoretical Model of behavior change (e.g., stages of action and maintenance) [47]. That is, COVID-19 or a similar pandemic would increase awareness of health-seeking behavior such as exercise, and further facilitate the motivation to initiate and maintain the behavior. Therefore, public health policy-makers should not only consider strategies to encourage exercise before a pandemic, but also take this pandemic as an opportunity to promote exercise for future similar pandemics.

### 4.2. Exercise and Mood

We observed that exercise frequency both pre and during the COVID-19 pandemic impacts mood states. Generally, higher exercise frequency during COVID-19 was associated with better mood states, while the effect of prepandemic exercise frequency on mood states was moderated by the change in exercise frequency (before vs. during). Specifically, those who frequently exercised before the pandemic (i.e., exercised for 2–3 days or four days or more) experienced a significant decline in their mood states when they decreased their exercise frequency during the pandemic.

These results are in line with other research on the positive psychological benefits of exercise. For example, a meta-analytic study indicated an association between exercise and improved mood states, with an overall effect size of 0.24 and a mean change effect size of 0.38 for the treatment versus the control group and pretest-posttest studies, respectively [48]. Frequent exercise or physical activity might lead to adaptation of biological systems, including changes in neural hormones and endorphins [49], increasing density and efficiency of mineralocorticoid receptors and diminishing the cortisol synthesis [50], as well as improving cardiorespiratory fitness and strength [51]. Furthermore, exercise also elevates self-efficacy, self-esteem, feelings of mastery [51,52,53], and cognitive function [54,55,56]. These positive physiological and psychological changes might be the mechanisms for improvements in mood states associated with both exercise before and during the pandemic.

Notably, higher total POMS scores reflect either higher positive mood states (e.g., vigor) or less negative mood states (e.g., depression/anxiety, fatigue, or irritability). Research has shown that the ratings of the arousing emotional pictures were significantly decreased after an exercise session [57], suggesting exercise reduces anxiety and increases resilience toward emotional stressors. Additionally, alleviation of negative mood states [58], a decrease in trait anxiety [59,60], and a decrease in the emergence of depression [61] have also been observed with exercise. Based on the cross-stressor adaptation hypothesis [62], exercise might result in the adaptation of the sympathetic nervous system and the hypothalamus–pituitary–adrenal axis [49,63,64], which in turn lead to anxiolytic effects. This suggests that frequent exercisers might benefit from anxiolytic effects of exercise, especially during the COVID-19 pandemic [65,66,67].

It should be noted that prepandemic frequent exercisers (e.g., frequency of four days or more) experienced worse mood states if they decreased their exercise frequency during the pandemic. Decrease in exercise levels, known as detraining [68], can have adverse effects both on exercise-induced physiological adaptation [69,70], as well as psychological adaptations [71]. Other studies have shown that the cessation of regular exercise is linked to negative mood states [72,73], as well as increases in somatic depressive symptoms (e.g., fatigue, Berlin, Kop, and Deuster [72]). It is possible that a decrease in cardiorespiratory fitness [72], self-efficacy [51,74], or changes in obligatory exercise beliefs [75] contributed to declines in mood states when frequent exercisers decreased their exercise frequency during the COVID-19 pandemic. In sum, maintenance of exercise behavior patterns, particularly in the active population, is essential to preserve mood states during the COVID-19 pandemic or any similar future pandemics.

### 4.3. Limitation and Future Directions

Some drawbacks of the study should be acknowledged. The translated questionnaires used in this study were not validated due to time limitations. In order to conduct this study during the brief window of governmental recommendations and restrictions related to COVID-19, the questionnaires were translated from English into 18 different languages including traditional Chinese for use in Taiwan, resulting in the lack of time to proceed with the validation of the questionnaires as well as cultural adaptations. An additional limitation is the cross-sectional design of the study and the use of self-report data. However, the “IRG on COVID and exercise study” is planning to conduct a second wave after the cessation of the COVID-19 pandemic to investigate the trends of exercise behavior and mood states of the respondents over time. Finally, collecting data through the internet might potentially limit the participation of certain groups (e.g., individuals who do not have internet access). However, the internet penetration rate in Taiwan reached 89.6% in 2019, and the internet access rate for individuals aged 12 and above is as high as 88.8% [76], suggesting the high accessibility and usage of the internet by Taiwanese.

Our findings indicate that exercise behavior before a pandemic outbreak is likely to affect exercise behavior and mood states during a pandemic. Specifically, the results show that age, gender, and educational level played a role in exercise behavior change during the pandemic. Therefore, exercise professionals and policy-makers should work together to provide comprehensive and practical strategies specifically for the populations who are at risk of decreasing their exercise frequency before a similar pandemic in the future. For example, exercise training sessions can shift from in-person to online settings [77]. This shift might require exercise practitioners acquiring several new skills such as online communication strategies, online exercise program design, and online evaluation of the clients’ progress. Additionally, sending motivational messages through the internet, such as EdTech [78] and social media (e.g., Facebook, Wojcicki et al. [79]), could be incorporated to promote exercise participation.

## 5. Conclusions

Our study is the first study conducted to investigate the changes in exercise behavior and mood states during the COVID-19 pandemic in Taiwan. The majority of Taiwanese were able to maintain their exercise behavior with respect to frequency, intensity, and duration during the COVID-19 pandemic, and predictive data shows that they are also likely to maintain their exercise frequency during a future similar pandemic. Additionally, the mood state was affected by exercise frequency both during and before the pandemic, and it was negatively impacted if active individuals decreased their exercise frequency during the pandemic. This study thus provides evidence of the importance of promoting exercise both before and during a pandemic.

## Figures and Tables

**Table 1:** Participants’ demographic data (*n* = 1114).

Factor Levels	*n*	% Female	*M*_age_	*SD*_age_
**Overall**	1114	53.9	35.9	15.16
**Gender**				
Female	601		35.2	15.31
Male	457		37.5	14.65
Other	56		30.9	16.21
**Income**				
Low	229	60.3	25.2	12.44
Medium	706	54.7	37.7	14.31
High	146	44.5	45.7	12.46
Missing	33	57.6	26.7	16.13
**Education**				
Less than high school High school graduate or GED Completed vocational school or college Some vocational school or college Some graduate school Master’s degree Doctoral degree Missing	104 102 370 48 108 280 101 1	69.2 48.0 55.9 60.4 55.6 53.2 33.7 100	16.0 24.6 37.9 30.8 34.4 41.7 48.7 13.0	3.6 13.5 14.6 18.4 12.6 10.4 10.2 NA
**Living environment**				
Urban/Suburban	1026	54.4	36.42	15.14
Rural	86	48.8	29.51	13.97
Missing	2	50	43.00	14.14

Note: The variable income was categorized into three groups in response to the question, “compared with the average income in your country, which one would you say is your household income?”; GED = General Educational Development. NA = nonavailable.

**Table 2:** Participants’ report on governmental restrictions and recommendations, and the status of the recreational facilities in Taiwan vs. international data.

Data	Yes	No	Missing
**Restrictions**			
Taiwan	7.5%	92.4%	0.2%
International	79.7%	20.3%	0.1%
**Recommendations**			
Taiwan	81.8%	18.2%	0%
International	85.3%	14.5%	0.2%
**Gyms closed**			
Taiwan	34.2%	65.4%	0.4%
International	91.1%	8.8%	0.1%
**Outdoor facilities closed**			
Taiwan	9.5%	90.2%	0.3%
International	76.3%	23.3%	0.4%

Note: Participants were asked: “do you live under any type of socially limiting formal restrictions that were established by your government or regional/local authorities?”; “are there recommendations from governmental, regional, or local authorities regarding ‘social distancing’ or ‘social isolation’ where you live?”.

**Table 3:** Change in exercise behavior. Statistical results for the cumulative link models (CLM) predicting exercise frequency during a pandemic with prepandemic exercise frequency and different covariates in separate models.

Coefficients	Estimate (*SE*)	*p*-Value
**Model: Exercise pre**		
Exercise_pre2-1_	1.95 (0.17)	<0.001
Exercise_pre3-2_	3.01 (0.17)	<0.001
**Model: Exercise pre × Gender**		
Exercise_pre2-1_	1.51 (0.26)	<0.001
Exercise_pre3-2_	3.30 (0.29)	<0.001
Gender Female	−0.20 (0.13)	0.12
Gender Male	−0.10 (0.13)	0.43
Gender Other	0.30 (0.21)	0.16
Exercise_pre2-1_ × Female	0.63 (0.29)	0.03
Exercise_pre3-2_ × Female	−0.38 (0.31)	0.22
Exercise_pre2-1_ × Male	0.28 (0.31)	0.36
Exercise_pre3-2_ × Male	−0.28 (0.31)	0.36
Exercise_pre2-1_ × Other	−0.91 (0.48)	0.06
Exercise_pre3-2_ × Other	0.66 (0.53)	0.21
**Model: Exercise pre × Age**		
Exercise_pre2-1_	2.39 (0.45)	<0.001
Exercise_pre3-2_	2.65 (0.44)	<0.001
Age	0.01 (0.00)	<0.01
Exercise_pre2-1_ × Age	−0.02 (0.01)	0.23
Exercise_pre3-2_ × Age	0.01 (0.01)	0.34
**Model: Exercise pre × Education**		
Exercise_pre2-1_	2.41 (0.26)	<0.001
Exercise_pre3-2_	3.16 (0.24)	<0.001
Education 1	−0.14 (0.28)	0.60
Education 2	0.10 (0.16)	0.52
Education 3	−0.02 (0.22)	0.94
Education 4	0.01 (0.14)	0.93
Education 5	−0.08 (0.45)	0.86
Education 6	0.09 (0.23)	0.70
Education 7	0.04 (0.22)	0.86
Exercise_pre2-1_ × Education 1	0.57 (0.73)	0.44
Exercise_pre3-2_ × Education 1	−0.07 (0.48)	0.89
Exercise_pre2-1_ × Education 2	−0.64 (0.37)	0.09
Exercise_pre3-2_ × Education 2	−0.05 (0.34)	0.89
Exercise_pre2-1_ × Education 3	−0.74 (0.57)	0.19
Exercise_pre3-2_ × Education 3	−0.22 (0.45)	0.62
Exercise_pre2-1_ × Education 4	−0.88 (0.34)	<0.01
Exercise_pre3-2_ × Education 4	−0.33 (0.32)	0.29
Exercise_pre2-1_ × Education 5	1.93 (1.00)	0.05
Exercise_pre3-2_ × Education 5	0.75 (1.00)	0.45
Exercise_pre2-1_ × Education 6	−0.49 (0.56)	0.38
Exercise_pre3-2_ × Education 6	0.49 (0.52)	0.35
Exercise_pre3-2_ × Education 6	0.26 (0.52)	0.62
Exercise_pre2-1_ × Education 7	−0.56 (0.55)	0.31
**Model: Income**		
Income Medium	0.15 (0.14)	0.28
Income High	0.43 (0.20)	0.03
**Model: Exercise pre × Income**		
Exercise_pre2-1_	1.81 (0.37)	<0.001
Exercise_pre3-2_	3.47 (0.38)	<0.001
Income Medium	0.18 (0.17)	0.30
Income High	0.23 (0.26)	0.37
Exercise_pre2-1_ × Income Medium	0.11 (0.42)	0.80
Exercise_pre3-2_ × Income Medium	−0.51 (0.41)	0.22
Exercise_pre2-1_ × Income High	0.25 (0.65)	0.70
Exercise_pre3-2_ × Income High	−1.81 (0.53)	0.13

Note: Prepandemic exercise levels: 1 = “1 day or less”; 2 = “2–3 days”; 3 = “4 days or more”. Education levels: 1 = “Doctoral degree”, 2 = “Master’s degree”, 3 = “Some graduate school”, 4 = “Completed vocational school or college”, 5 = “Some vocational school or college”, 6 = “High school graduate or GED”, 7 = “Less than high school.

**Table 4:** Exercise frequency and change in mood states. Statistical results for the linear model analyzing mood states (POMS) with exercise frequency pre and during the pandemic.

Coefficients	Estimate (*SE*)	*p*-Value
**Model: Exercise pre × Exercise during**		
Intercept	3.61 (0.03)	<0.001
Exercise_pre2-1_	−0.04 (0.07)	0.55
Exercise_pre3-2_	−0.16 (0.07)	0.03
Exercise_during2-1_	0.29 (0.07)	<0.001
Exercise_during3-2_	0.14 (0.07)	0.04
Exercise_pre2-1_ × Exercise_during 2-1_	0.32 (0.13)	0.01
Exercise_pre3-2_ × Exercise_during 2-1_	0.42 (0.19)	0.03
Exercise_pre2-1_ × Exercise_during 3-2_	−0.48 (0.19)	0.01
Exercise_pre3-2_ × Exercise_during 3-2_	0.33 (0.14)	0.02

Note: Exercise levels pre and during the pandemic: 1 = “1 day or less”; 2 = “2–3 days”; 3 = “4 days or more”.

**Table 5:** Post-hoc contrasts comparing mood states with the pairwise test for different exercise frequency groups pre and during the pandemic.

Contrast	Estimate (*SE*)	*p*-Value
**Exercise before = 1 day or less**		
Exercise during: 1 day or less–2–3 days	0.07 (0.10)	0.50
Exercise during: 1 day or less–4 days or more	−0.28 (0.13)	0.07
Exercise during: 2–3 days–4 days or more	−0.35 (0.15)	0.07
**Exercise before = 2–3 days**		
Exercise during: 1 day or less–2–3 days	−0.25 (0.08)	<0.01
Exercise during: 1 day or less–4 days or more	−0.12 (0.13)	0.53
Exercise during: 2–3 days–4 days or more	0.13 (0.12)	0.53
**Exercise before = 4 days or more**		
Exercise during: 1 day or less–2–3 days	−0.67 (0.18)	<0.001
Exercise during: 1 day or less–4 days or more	−0.88 (0.13)	<0.001
Exercise during: 2–3 days–4 days or more	−0.20 (0.08)	0.01

Note: *p*-value adjustment: Holm method.

## Data Availability

Data are available upon request.
